# Gene Expression Profiling during Pregnancy in Rat Brain Tissue

**DOI:** 10.3390/brainsci4010125

**Published:** 2014-03-04

**Authors:** Phyllis E. Mann

**Affiliations:** Department of Biomedical Sciences, Cummings School of Veterinary Medicine, Tufts University, 200 Westboro Rd., N. Grafton, MA 01536, USA; E-Mail: phyllis.mann@tufts.edu; Tel.: +1-508-887-4911; Fax: +1-508-839-8740

**Keywords:** PCR array, maternal behavior, pregnancy, gene expression, brain

## Abstract

The neurophysiological changes that occur during pregnancy in the female mammal have led to the coining of the phrases “expectant brain” and “maternal brain”. Although much is known of the hormonal changes during pregnancy, alterations in neurotransmitter gene expression have not been well-studied. We examined gene expression in the ventromedial nucleus of the hypothalamus (VMH) during pregnancy based on the fact that this nucleus not only modulates the physiological changes that occur during pregnancy but is also involved in the development of maternal behavior. This study was designed to identify genes that are differentially expressed between mid- and late-pregnancy in order to determine which genes may be associated with the onset and display of maternal behavior and the development of the maternal brain. A commercially available PCR array containing 84 neurotransmitter receptor and regulator genes (RT^2^ Profiler PCR array) was used. Brains were harvested from rats on days 12 and 21 of gestation, frozen, and micropunched to obtain the VMH. Total RNA was extracted, cDNA prepared, and SYBR Green qPCR was performed. In the VMH, expression of five genes were reduced on day 21 of gestation compared to day 12 (*Chrna6*, *Drd5*, *Gabrr2*, *Prokr2*, and *Ppyr1*) whereas *Chat*, *Chrm5*, *Drd4*, *Gabra5*, *Gabrg2*, *LOC289606*, *Nmu5r2*, and *Npy5r* expression was elevated. Five genes were chosen to be validated in an additional experiment based on their known involvement in maternal behavior onset. This experiment confirmed that gene expression for both the CCK-A receptor and the GABA_A_R γ2 receptor increases at the end of pregnancy. In general, these results identify genes possibly involved in the establishment of the maternal brain in rats and indicate possible new genes to be investigated.

## 1. Introduction

The physiological changes that occur during pregnancy in the female mammal include neural adaptations, which have led to the coining of the phrases “expectant brain” or “maternal brain” [1,2]. These adaptations include changes in stress responsiveness [3], learning and memory [4], preparation for parturition and lactation with alterations in oxytocin and prolactin regulation [5], respectively, and changes in feeding behavior and energy metabolism in favor of the fetus [6,7]. Many of these changes involve the ventromedial nucleus of the hypothalamus (VMH) (e.g., [8]).

The VMH is also involved in a variety of reproductive behaviors, including sex behavior [9] and the onset of maternal behavior [10,11]. The role of the VMH in the onset of maternal behavior has been shown to be an inhibitory one. While spontaneous maternal behavior is displayed during the periparturitional period [12,13], maternal behavior does not occur spontaneously in virgin rats. However, if virgin females are housed with young pups for several days, they can be induced or “sensitized” to show maternal behavior with an average latency of 5–7 days [14]. Paradoxically, spontaneous maternal behavior does occur in both female and male 20–24-day-old juvenile rats [15,16] and appears to be “hard-wired”, and not dependent on the endocrine system [15,17]. The reason that juvenile maternal behavior disappears and adult virgin rats are not spontaneously maternal is due to the existence of a neural circuit that actively inhibits the display of maternal behavior. Early studies have shown that rendering the virgin female anosmic shortens the latencies to become maternal to 2–3 days [18]. Further studies demonstrated that both the main and accessory (vomeronasal) olfactory systems are involved in the inhibition of maternal behavior [18,19], as well as the medial amygdala (meA) and bed nucleus of the stria terminalis (BnST) [20,21,22]. Additional evidence indicates that the hypothalamus also contributes to the maternal behavior inhibitory neural circuit. Lesioning the VMH decreases the latency to display maternal behavior in virgin rats and in first-time pregnant rats [10,11].

Therefore, the objective of the current study was to identify neurotransmitter and neuromodulator genes that may be change from mid-pregnancy (day 12) to late pregnancy (day 21) for the purpose of determining which genes may regulate the changes in the expectant or maternal brain.

## 2. Results and Discussion

**Figure 1:**
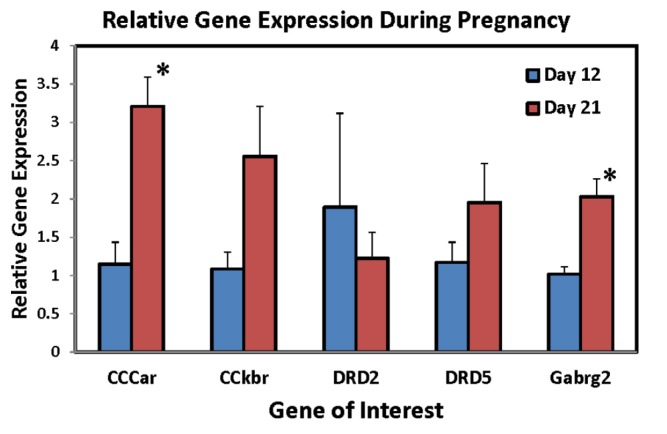
Relative mRNA expression levels of *CCKar*, *CCKbr*, *Drd2*, *Drd5*, and *Gabrg2* genes in the ventromedial nucleus of the hypothalamus (VMH) following RT-PCR in primigravid rats on either day 12 (*N* = 5/group) or day 21 (*N* = 6/group) of gestation. * Significantly different from day 12 of gestation, *p* < 0.05).

Table 1 lists the genes that displayed at least a two-fold difference between groups, either decreasing (Part A) or increasing (Part B) in the VMH at the end of pregnancy. Further validation was performed on five genes. In the VMH, RT-PCR using 5–6 animals per group confirmed that primigravid rats on day 21 of gestation had significantly higher CCK-A receptor expression levels than day 12 of gestation (*p* = 0.0024, Figure 1). CCK-B receptor levels upon validation not only did not decrease as preliminary results indicated, but increased, approaching significantly higher levels at the end of pregnancy (*p* = 0.08, Figure 1). Expression levels in both dopamine D2 and D5 receptors did not change over the course of pregnancy contrary to the array results (*p*’s > 0.05, Figure 1). GABA(A) receptor levels, on the other hand, were confirmed demonstrating a significant increase at the end of pregnancy (*p* = 0.005, Figure 1).

**Table 1:** (**A**) Genes that Decrease at the End of Pregnancy; (**B**) genes that Increase at the End of Pregnancy.

**A**	**Fold Difference**
Acetylcholinesterase (*Hache*)	2.92
Cholinergic receptor, muscarinic 1 (*Chrm1*)	10.99
Cholinergic receptor, muscarinic 3 (*Chrm3*)	22.56
Cholinergic receptor, muscarinic 4 (*M4*)	2.84
Cholinergic receptor, nicotinic, alpha polypeptide 2 (*ACHR*)	3.59
Cholinergic receptor, nicotinic, beta polypeptide 2 (*Chrnb2*)	2.08
Cholecystokinin B receptor (*CHOLREC*)	2.1
Dopamine receptor D5 (*Drd5*)	3.57
G protein-coupled receptor 83 (*Gir*)	5.53
Gastrin releasing peptide receptor (*Grpr*)	4.29
Glutamic acid decarboxylase 1 (*Gad67*)	2.83
Neuropeptide FF receptor 1 (*Gpr147/NPFF1*)	8.95
Neuropeptide Y receptor Y1 (*NPY-1*)	5.42
Prokineticin receptor 2 (*Gpr73l1*)	5.83
Prolactin releasing hormone receptor (*Gpr10/Uhr-1*)	8.22
Gamma-aminobutyric-acid receptor alpha-2 subunit precursor (GABA(A) receptor) (*LOC289606*)	2.1
Somatostatin receptor 1 (*Gpcrrna*)	3.65
Somatostatin receptor 3 (*Smstr28*)	3.29
Somatostatin receptor 4 (*Smstr4*)	3.19
Tachykinin receptor 1 (*Tac1r*)	3.54
**B**	**Fold Difference**
Cholecystokinin A receptor (*Cck-ar*)	2.64
Dopamine receptor 2 (*dopamine D2*)	2.67
Dopamine receptor D1A (*D1a/Drd-1*)	2.1
Gamma-aminobutyric acid A receptor, gamma 2 (*Gabrg2*)	2.24
Neuropeptide FF receptor 2 (*Gpr74/Npff2*)	3.09

The modifications that occur in the brain during pregnancy presumably prepare the female rat for the physiological and behavioral changes necessary for motherhood. Reproductive experience, therefore, can be considered similar to the changes that occur in utero and during puberty on sexual differentiation of the brain [23]. The use of a Neurotransmitter Receptors and Regulators PCR Array allows the researcher to confirm the changes in the brain during pregnancy that are already described and to discover possible new genes involved with these changes. Alterations in estrogen, progesterone, prolactin, and oxytocin during pregnancy are well-known [24] and prepare the female physiologically and behaviorally for the requirements postpartum. The role of neurotransmitters is less understood. The present study has identified genes in the VMH that change during pregnancy that have not been reported previously. The most surprising finding, in fact, was the number of cholinergic genes that decreased at the end of pregnancy (Table 1). While much is known of the changes in the cholinergic system during fetal development [25,26], very little is known of its role in the maternal brain during pregnancy. In sheep and goats, the cholinergic system seems to regulate maternal recognition of offspring and maternal attachment after parturition [27]. In the VMH, specifically, infusion of acetylcholine or its agonists into the VMH stimulates lordosis behavior in female rats [28] while* in vitro* administration to VMH explants increases single unit activity [29,30]. Rainbow* et al.*, [31] showed that estradiol increased the levels of muscarinic receptors in the VMH and correlated these results with lordosis behavior. Even though the changes in cholinergic genes were not confirmed, together this evidence indicates the possible role for cholinergic neurons in the VMH in the regulation of another reproductive behavior, maternal behavior, and points to a new avenue of research involving the cholinergic system in pregnancy-induced modifications of the brain.

The change in dopamine levels in the brain during pregnancy has also not been well-studied. Before day 12 of pregnancy there is a down regulation of the dopamine system in the hypothalamus that allows for the diurnal and nocturnal surges of prolactin of early pregnancy in the rat [32]. Studies have shown a reduction in tuberinfundibular dopamine (TIDA) activity at this time to allow for the surges [33], but not much else is known until late pregnancy when it thought that a reduction in dopamine activity might be partially involved in the prepartum prolactin surge [34]. There are also changes in dopamine receptor concentrations in the brain during pregnancy. Bakowska and Morrell, 1995 [35] choosing areas that might be relevant to maternal behavior onset, found that both D1 and D2 dopamine receptors were reduced in the striatum and nucleus accumbens in late pregnancy. Studies on the dopaminergic regulation of maternal behavior have shown that systemic administration of dopamine antagonists such as haloperidol (D1/D2, [36]), SKF83566 (D1, [37]), or pimozide (D2, [37]) interfere with maternal responding during lactation. When dopamine receptor antagonists are injected intracranially, drugs that are selective for the DA D1 receptor are more efficacious than DA D2 antagonists into the preoptic area (SCH23390, D1 [38]) or nucleus accumbens ([39] but see [40]). Less is known of dopamine’s role in the onset of maternal behavior. Hansen *et al.*, 1991 [41] found that depletion of dopamine using 6OHDA injections into the ventral tegmental area during pregnancy completed disrupted maternal behavior postpartum. Using the pregnancy terminated model, studies indicate that DA D1 receptors are involved in the onset of maternal behavior when infused into the nucleus accumbens [42,43]. The results of the PCR array in the present study indicate a decrease at the end of pregnancy in the dopamine D5 receptor (part of the D1-like family) gene and an increase in dopamine D2 and D1A receptor expression at the end of pregnancy. However, the RT-PCR results in the follow-up validation experiment did not significantly confirm these changes in the VMH. It is possible that the validation study was underpowered or that the differences in probe handling, RNA extraction and conditions between the two techniques affected the results. Further studies are needed to pursue the role of dopamine in the maternal brain during pregnancy. The inhibitory neurotransmitter, GABA, acts at GABA receptors to mediate most inhibitory signaling in the brain. The GABA_A_R is an ion-gated chloride channel that has a pentameric complex usually consisting of 2 α (1–6) and 2 β (1–3) subunits with an additional γ (1–3), δ, or ρ subunit. The GABA_A_R γ2 is thought to be present at postsynaptic membranes mediating inhibitory signaling. The PCR array and subsequent validation experiment confirmed the increase in GABA_A_R γ2 mRNA in the VMH at the end of pregnancy. These results, however, are in contrast with the decrease in GABA_A_R γ2 mRNA that occurs in the cerebral cortex and hippocampus across pregnancy [44], and different from Fenelon and Herbison, 1996 [45] who report no change in GABA_A_R γ2 mRNA in the paraventricular nucleus (PVN) or supraoptic nucleus (SON) in the hypothalamus at the end of pregnancy. The role of the GABA_A_R, in general, in the onset of maternal behavior is not well studied. Carretero *et al.*, 2003 [46] found that administration of bicuculline, a GABA_A_R receptor antagonist, into the accessory olfactory bulb stimulates the onset of maternal behavior in virgin rats, but no studies have examined the role of the GABA_A_R on the onset of maternal behavior at parturition. During pregnancy, however, research indicates a possible role for the GABA_A_R γ2 in neurosteroid binding [47] and, since the GABA_A_R γ2 binds the benzodiazepines, there may be a role in pregnancy-induced stress hyporesponsiveness [48].

Cholecystokinin (CCK) is a gut peptide that is released postprandially in the proximal small intestine [49]. In addition CCK is found in the brain and is involved in feeding behavior (a satiety factor), pain and analgesia, and learning and memory (for review see [50]). During pregnancy, the satiety effect of CCK is absent or diminished. Ladyman *et al.*, 2011 [51] found that day 14 pregnant rats did not reduce food intake after injections of CCK-8 compared to nonpregnant controls. The actions of CCK are mediated by Type A and Type B CCK receptors with Type A mostly present in the periphery and select CNS sites and Type B throughout the central nervous system [52], although the satiety actions of CCK is thought to be mediated by CCK-A receptors. The role of CCK in the onset of maternal behavior has been equivocal. Linden *et al.*, 1989 [53] demonstrated a fast onset of maternal behavior in ovariectomized, estrogen-treated virgin rats, however, Mann *et al.*, 1995 [54] found no effect of CCK in stimulating maternal behavior in virgin rats, but did find that CCK antagonists disrupted maternal behavior in postpartum, lactating rats. In the VMH specifically, CCK seems to have an inhibitory role in neuronal firing [55] and its binding influenced by the presence of estradiol [56]. In the present study, the validation experiment replicated the finding that there is an increase in CCK-A receptors mRNA on day 21 of pregnancy as compared to day 12. CCK-B receptor results however were not confirmed. In fact, the validation experiment showed a nonsignificant increase in CCK-B receptor mRNA at the end of pregnancy. Further studies need to be performed to confirm these results.

## 3. Experimental Section

### 3.1. Animals

Nulliparous Sprague-Dawley female rats (225–250 g; CRL:CD(SD)BR) were purchased from Charles River Laboratories, Inc. (Kingston, NY, USA). The females were triply-housed in polypropylene cages (45 × 25 × 20 cm) with food and water were available *ad libitum* in light (on 0500–1900 h)- and temperature (21–25 °C)-controlled rooms. All animals were maintained in accordance with the guidelines of the Committee on the Care and Use of Laboratory Animal Resources, National Research Council.

One week after the experimental animals arrived, they were housed with experienced males. The day that sperm were present in the vaginal lavage was considered day 1 of pregnancy.

### 3.2. Tissue Collection

On either days 12 or 21 of gestation, between 0900 and 1200 h, rats were briefly anesthetized with CO_2_ and brains were removed rapidly and flash-frozen in methylbutane (−15 °C) and buried in dry ice. Brains were then kept at −80 °C until micropunched in a cryostat (−20 °C). Micropunches using the atlas of Paxinos and Watson [57] were collected from the VMH (1 mm in diameter by 1 mm deep; from ≈ −2.04 to ≈ −3.04 mm from Bregma, between midline and ≈ 1 mm lateral and ≈ 9 to 10 mm ventral to the dura) for PCR Array analysis. Samples were immediately stored at −80 °C prior to processing.

Five genes were chosen to be validated based on having more than 2-fold differences in gene expression determined by the PCR array and their known involvement in the regulation of maternal behavior. An additional group of animals (*N* = 5–6/group) were treated as described above.

### 3.3. Sample Preparation

Total RNA was extracted from micropunched tissues using the RNeasy Mini Kit (Qiagen, Valencia, CA, USA) following the manufacturer’s instructions. cDNA was synthesized by RT^2^ First Strand Kit (Qiagen) following the manufacturer’s instructions.

### 3.4. Quantitative Real-Time PCR (qPCR)

Relative mRNA expression of 84 neurotransmitter receptors and regulators were determined using the rat Neurotransmitter Receptors and Regulators PCR Array (PARN-060A, SABiosciences, for a complete list of genes, see [58]). Quantitative real-time PCRs were performed on an Applied Biosystems 7500 (Foster City, CA, USA) using RT^2^ Real-Time SYBR green PCR master mix. Expression of each gene was normalized using the mean expression of five housekeeping genes. Data from the day 21 group was compared using the mean data from the day 12 group (control) as the calibrator. Linearized relative expression was obtained according to the 2^−ΔΔCT^ method [59]. Genes that had positive or negative fold changes of more than two were considered candidates for validation.

### 3.5. Reverse Transcription PCR (RT-PCR)

The oligonucleotide sequences used for RT-PCR are presented in Table 2 and were designed using Primer Express (Applied Biosystems) and purchased from Integrated DNA Technologies. PCR amplification was performed using a Thermal Cycler (BioRad, Hercules, CA, USA) with the following reaction profile: 94 °C for 2 min, 94 °C for 45 s, 55 °C for 45 s, 72 °C for 1 min, 72 °C for 5 min for 30 cycles. The reaction mixture (25 μL) included 1 μL template DNA, 2.5 μL Platinum^®^ Taq polymerase (Applied Biosystems), 3.3 mM magnesium chloride, 0.5 μL of 10× dNTP and 5 μM of each primer. PCR products were checked on a 2.2% agarose gel using ethidium bromide.

**Table 2:** Oligonucleotide sequences for primers used in the RT-PCR validation.

Gene	Accession #	Direction	Sequence
Colecystokinin A receptor (*CCKar*)	NM_012688	Forward	5'- ATGCAGCAGTCCTGGCAAACATTC-3'
		Reverse	5'-TTTGGCAGATTTCTTCTGGCTGGC-3'
Colecystokinin B receptor (*CCKbr*)	NM_013165	Forward	5'-AGCGATACAGCGCCATCTG-3'
		Reverse	5'-CGTGGGAGCGTGTTTGC-3'
Dopamine receptor 2 (*Drd2*)	NM_012547	Forward	5'-TGACAGTCCTGCCAAACCAGAGAA-3'
		Reverse	5'-TGGGCATGGTCTGGATCTCAAAGA-3'
Dopamine receptor 5 (*Drd5*)	NM_012768	Forward	5'-TGTGTATCATCAGCGTGGACCGTT-3'
		Reverse	5'-ATTGAGTTGGACCGGGATGAAGGA-3'
Gamma-aminobutyric acid A receptor (*Gabrg2*)	NM_183327	Forward	5'-GCAACCGGAAACCAAGCAAGGATA-3'
		Reverse	5'-GGTGGGTGGCATTGTTCATTTGGA-3'

### 3.6. Data Analysis

The RT-PCR data comparing days 12 and 21 of gestation were analyzed with two-sample *t*-tests. Differences were considered significant if *p* < 0.05.

## 4. Conclusions

In summary, the combination of gene expression profiling and subsequent validation experiments have confirmed changes in mRNA for neurotransmitter known to be involved with the physiological changes during pregnancy and the onset of maternal behavior. In addition the use of PCR arrays during pregnancy has ascertained several new genes that may be important for the development of the maternal brain.
